# SmartFilm Tablets for Improved Oral Delivery of Poorly Soluble Drugs

**DOI:** 10.3390/pharmaceutics14091918

**Published:** 2022-09-10

**Authors:** Ayat Abdelkader, Eduard Preis, Cornelia M. Keck

**Affiliations:** 1 Department of Pharmaceutics and Biopharmaceutics, Philipps-Universität Marburg, Robert-Koch-Str. 4, 35037 Marburg, Germany; 2 Assiut International Center of Nanomedicine, Al-Rajhi Liver Hospital, Assiut University, Assiut 71515, Egypt

**Keywords:** paper, granules, oral drug delivery, tablet manufacturing, ex vivo porcine intestinal model, intestinal permeability

## Abstract

(1) Background: Numerous oral drugs exhibit limited bioavailability due to their poor solubility and poor intestinal permeability. The smartFilm technology is an innovative approach that improves the drug aqueous solubility via incorporating the drug in an amorphous state into a cellulose-based matrix, i.e., paper. smartFilms can be transformed into a free-flowing physical form (i.e., paper granules) that can be compressed into tablets with optimum physico-chemical and pharmaceutical properties. The aim of this study was to investigate if smartFilm tablets are suitable for improved oral delivery of poorly water-soluble drugs. (2) Methods: Curcumin is a poorly soluble drug with low intestinal permeability and was used for the production of curcumin-loaded smartFilms. The curcumin-loaded smartFilms were transferred into smartFilm granules which were then compressed into curcumin-loaded smartFilm tablets. The tablets were characterized regarding their physico-chemical and pharmaceutical properties, and the intestinal permeability of curcumin was determined with the ex vivo porcine intestinal model. The ex vivo intestinal permeability of curcumin from the smartFilm tablets was compared to a physical mixture of curcumin and paper and to a classical and to an innovative commercial product, respectively. (3) Results: The produced curcumin-loaded smartFilm tablets fulfilled the European Pharmacopoeia requirements, incorporated curcumin in amorphous state within the cellulose matrix and exhibited an enhanced dissolution rate. The ex vivo intestinal permeation data were shown to correlate to the in vitro dissolution data. The ex vivo intestinal permeation of curcumin from the smartFilm tablets was about two-fold higher when compared to the physical mixture and the classical commercial product. No differences in the ex vivo bioavailability were found between the smartFilm tablets and the innovative commercial product. (4) Conclusions: smartFilm tablets are a cost-effective and industrially feasible formulation approach for the formulation of poorly water-soluble drugs, i.e., BCS class II and IV drugs.

## 1. Introduction

Oral delivery is the most preferred route for drug administration [1]. The efficient delivery of oral drugs to the systemic circulation is primarily affected by the physico-chemical properties of the drug (e.g., solubility, stability) and/or the physiological properties of the gastrointestinal tract (GIT), e.g., the harsh acidic environment, the abundance of digestive enzymes, and the intestinal mucosal physical absorption barrier [2].

**Figure 1:**
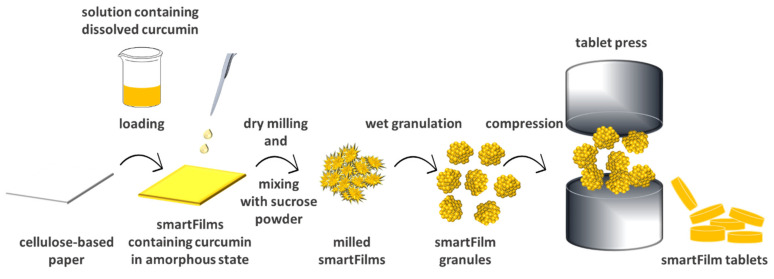
Scheme of production of curcumin-loaded smartFilm granules and smartFilm tablets.

Various approaches have been utilized to increase the poor aqueous solubility of drugs, including micelle formation, lipid-based formulations, nanoemulsions, self-emulsifying drug delivery systems, solid dispersions, inorganic nanocarriers, or nanocrystals [3,4,5]. Recently, smartFilms have emerged as novel oral delivery systems to improve the poor aqueous solubility of drugs [6]. The smartFilm technology utilizes the pores of cellulose matrices as loading sites in which the therapeutic agent is loaded in an amorphous form, resulting in enhancing the dissolution rate of the loaded drug. The production of smartFilms involves dissolving a poorly soluble drug in an appropriate solvent, applying the resulting solution on a porous cellulose-based paper, and drying the obtained drug-loaded smartFilms (Figure 1). The matrix of the paper retains the drug in an amorphous state, and thus improves its solubility [6,7]. Several studies highlight the smartFilm technology as a potential oral drug delivery system and were able to transform smartFilms into an appropriate oral dosage form (i.e., paper tablets), which can be conveniently administered. These tablets were manually produced, without the addition of any excipients and fulfilled all requirements of the European Pharmacopoeia [8,9,10].

Despite its proven effectiveness, still, the smartFilm technology remained unrecognized by the pharmaceutical industry, because a large-scale production of paper tablets from paper cut outs—due to the poor flowability of the paper cut outs—was not possible. To address this issue, unloaded smartFilms were transformed into a free-flowing physical form (i.e., paper granules), rendering them highly convenient for high-speed, large-scale tablet manufacturing [10,11].

The transfer of paper into paper granules is required to allow for a large-scale production of the tablets. However, this granulation process requires wetting steps [10,11]. These wetting steps are not critical for the production of non-loaded, i.e., drug free, paper tablets, but might be critical if drug-loaded smartFilms—that contain drug in amorphous form—are transferred into tablets. The wetting might cause a (partial) dissolution of the drug during the wetting process and a subsequent re-crystallization upon the drying. The changes in crystalline state can then affect the dissolution velocity of the drug and—consequently—the oral bioavailability of the drug that was loaded into the smartFilms.

The aim of this study was therefore to investigate (i) if drug-loaded smartFilms can be transferred into smartFilm granules and smartFilm tablets and (ii) if these smartFilm tablets can maintain the amorphous state of the incorporated drug, which then should result in an improved dissolution rate and in an enhanced intestinal permeability of the poorly-water soluble drug.

Curcumin is a natural compound that can be obtained from the rhizome of the turmeric plant (*Curcuma longa* L.) and has been widely used as a preventive and therapeutic agent against numerous diseases [12]. This can be attributed to the endogenous pharmacological properties of curcumin, which include antioxidant, anti-inflammatory, antibacterial, antiviral, antihepatotoxic, antidepressant, and anticancer activities [13,14]. The biopharmaceutical classification system (BCS) classifies curcumin as class IV drug, indicating that curcumin is poorly soluble, with limited oral bioavailability and intestinal permeability [15]. These characteristics render curcumin the drug of choice for studying the impact of smartFilm tablet production via wet granulation on the dissolution rate and intestinal permeability of a poorly soluble drug.

The study was performed in two steps. In the first step, curcumin-loaded smartFilms were prepared and transferred into smartFilm granules. The granules were transformed into smartFilm tablets and their physico-chemical and pharmaceutical properties (e.g., thickness, content uniformity, mass uniformity, friability, hardness, disintegration time, and dissolution profile) were determined. The crystalline state of curcumin was also assessed and compared to that within the smartFilm granules and the smartFilm tablets (Figure 1). In the second step, the oral bioavailability, i.e., the intestinal permeability of curcumin, was determined ex vivo in a porcine intestinal model. The results obtained were compared to a physical mixture tablet, that contained the paper matrix and crystalline curcumin bulk powder material. In addition, the results were compared to two commercially available curcumin products. Commercial product I represented a classical formulation principle, i.e., it contained curcumin as raw powder in a hard capsule. Commercial product II contained “micellar curcumin”, i.e., curcumin and high concentrations of an o/w surfactant filled into a soft capsule. The surfactant solubilizes the lipophilic curcumin in micelles and thus provides excellent solubility [16]. Despite this, micellar curcumin was also found to be superior when compared to other drug delivery systems, i.e., oils, liposomes, phytosomes, cyclodextrines, or sub-micron particles. The major reason for this was attributed to its excellent digestive stability and increased post-digestive solubility, when compared to the other formulation strategies [16]. In fact, at present, the commercial product II, i.e., the “micellar curcumin”, is considered to be the most effective curcumin formulation available and was therefore selected as benchmark control for the curcumin-loaded smartFilm tablets.

## 2. Materials and Methods

### 2.1. Materials

Curcumin, which was used as *Curcuma longa* extract powder with a curcumin content of 80%, was obtained from Receptura Apotheke (Cornelius-Apothekenbetriebs-OHG, Frankfurt, Germany). Based on previous studies, commercially available, cellulose-based paper (Soft & Sicher, dm-drogerie markt GmbH + Co. KG, Karlsruhe, Germany) was used as paper matrix [8,10,17]. The paper was approved for food and skin contact and consisted of 100% fresh cellulose pulp that was made from four different tree species (Eucalyptus, Fagus, Picea and Pinus). Sucrose and sodium dodecyl sulfate (SDS) were purchased from Carl Roth GmbH + Co. KG (Karlsruhe, Germany). Anhydrous tribasic sodium phosphate was obtained from Fisher Scientific GmbH (Schwerte, Germany). Purified water was freshly obtained from a PURELAB Flex 2 (ELGA LabWater, Veolia Water Technologies GmbH, Celle, Germany). The commercial products were purchased from dm-Dorgeriemarkt (Marburg, Germany). Commercial product I—representing the classical formulation principle—was a hard capsule with 750 mg *Curcuma longa* extract powder that contained 35 mg curcumin. Each capsule also contained 2 mg piperine from black pepper and 25 µg cholecalciferol, along with hydroxypropyl cellulose and magnesium stearate as excipients. Commercial product II—representing the innovative formulation principle with excellent oral bioavailability—was a soft capsule with 40 mg *Curcuma longa* extract that contained 35 mg curcumin. Each capsule also contained 40 mg ascorbic acid and 5 µg cholecalciferol, along with polysorbate 80 and hydroxypropyl cellulose as excipients.

### 2.2. Methods

#### 2.2.1. Production of Curcumin-Loaded smartFilms and smartFilm Granules

Curcumin-loaded smartFilms were prepared as described previously [18], with slight modifications. Curcumin was dissolved in ethanol to produce a solution of 2.5 mg/mL. Small paper sheets (approx. 5 cm × 5 cm), with an individual mass of approximately 200 mg, were separately loaded with 0.5 mL of curcumin solution using an automatic micropipette. Following drying, the procedure was repeated several times to prepare 20 mg curcumin-loaded paper sheets (i.e., smartFilms with 10% (*w*/*w*) loaded curcumin). Subsequently, the dried curcumin-loaded smartFilms were used to prepare curcumin-loaded smartFilm granules.

The granules were prepared using the wet granulation method and purified water was used as a granulation liquid [11,19]. For this, the curcumin-loaded smartFilms were dry milled using a knife mill (Moulinex DP8108, Groupe SEB Deutschland GmbH, Frankfurt, Germany) for 1 min. Afterwards, sucrose was added to the blend to obtain milled smartFilms that contained 20% (*w*/*w*) sucrose [11]. The resulting mixture was slightly wetted by spraying purified water on top of the milled smartFilm/sucrose mixture. This was performed to dissolve the sucrose and to increase the density of the milled paper [11]. The wetted mixture was further grinded with the knife mill (1 min) and then transferred to a plastic sieve, where it was further wetted with purified water under shaking at 300 rpm (universal shaker SM-30 control, Edmund Bühler GmbH, Bodelshausen, Germany) for a period of 3–8 min to obtain the smartFilm granules [11]. The wet curcumin-loaded smartFilm granules were dried in the oven for 30 min at 120 °C (UN 30, Memmert GmbH + Co. KG, Schwabach, Germany). Afterwards they were sieved manually (mesh size 2.8 mm, Retsch GmbH, Haan, Germany) to obtain a size fraction ≤2.8 mm.

#### 2.2.2. Characterization of Curcumin-Loaded smartFilm Granules

The smartFilm granules were characterized regarding size and shape. In addition, bulk and tapped density and the angle of repose were determined according to previously described protocols [11]. Details to the methods used are given below.

##### Determination of Particle Size and Shape

The Feret’s diameter of the curcumin-loaded smartFilm granules was measured via digital image analysis using ImageJ software (National Institutes of Health, Bethesda, MD, USA) as described previously [20]. Ten representative images that contained approximately 200–300 granules were obtained with a Canon IXUS 190 digital camera (Canon Europe Ltd., Uxbridge, UK). The images were color-adjusted, and threshold analysis was performed to label the granules individually, then the Feret’s diameter of each granule was assessed by the software (Table S1). From the results obtained, the number based median particle size diameters d(n) 0.10, d(n) 0.50, d(n) 0.90, d(n) 0.95, and d(n) 0.99 were calculated. Furthermore, the sphericity of the granules was determined via calculating the aspect ratio as follows:

(1) $$\mathrm{Aspect}\;\mathrm{ratio}=\frac{d_{\max\left(\mathrm{Feret}\right)}}{d_{90{}^{\circ}\left(\mathrm{dmax}\right)}}$$

where *d*_max(Feret)_ is the maximum Feret’s diameter and *d*_90°(dmax)_ is Feret’s diameter perpendicular to *d*_max(Feret)_. An aspect ratio of 1.2 is usually accepted to describe spherical particles [21].

##### Determination of Bulk and Tapped Density

A mechanical tapping device (tap density tester TD200, Pharma Test Apparatebau AG, Hainburg, Germany), was used to determine the bulk and tapped density of curcumin-loaded smartFilm granules according to the test method 2.9.34. of the European Pharmacopeia [22]. Ten grams of the granules was placed into a 250 mL measuring cylinder. The starting volume and the final volume, after carrying out 10, 500, and 1250 taps on the same granules sample, were recorded and used to calculate the bulk and tapped density, respectively. Moreover, the flowability of the granules was determined from the tapped density (1250 taps) and bulk density via calculating Hausner’s ratio and the Carr’s index using the following equations [23]:

(2) $$\mathrm{Hausner}’s\;\mathrm{ratio}=\frac{\rho_{tapped}}{\rho_{bulk}}\;$$

(3) $$\mathrm{Carr}’s\;\mathrm{index}=100\left(\frac{\rho_{tapped-}\rho_{bulk}}{\rho_{tapped}}\right)\;$$

where ρ*_tapped_* is the tapped density and ρ*_bulk_* is the bulk density.

###### Angle of Repose

The angle of repose of curcumin-loaded smartFilm granules was determined using the flowability tester (Emmeram Karg Industrietechnik, Krailling, Germany) according to the test method 2.9.36. of the European Pharmacopeia [22]. Twenty grams of the granules was placed in the funnel of the tester. Then, the granules were stirred carefully to run through the funnel and accumulate on a fixed base to form a heap. The height of the granules heap was measured, and the angle of repose (α) was determined using the following equation:

(4) $$\tan\alpha =\frac{h}{0.5\;d_{b}}\;$$

where *h* is the height of the heap and *d*_b_ is the diameter of the base.

##### 2.2.3. Production of Curcumin-Loaded smartFilm Tablets

The smartFilm granules obtained were filled into the hopper of a single punch tablet press (EK0, Korsch GmbH, Berlin, Germany) and compressed with a compression force of 30 kN into flat-faced bevel-edged smartFilm tablets with a 10 mm flat-faced punch (Ritter Pharma-Technik GmbH, Stapelfeld, Germany). The properties of the produced tablets were assessed as described earlier [11], i.e., by subjecting the tablets to various tests, as described in the European Pharmacopeia [22]. Details to the methods used are given below.

##### 2.2.4. Characterization of Curcumin-Loaded smartFilm Tablets

###### Macroscopic and Microscopic Analysis

The smartFilm tablets obtained were first inspected visually and were then analyzed by scanning electron microscopy (SEM, Hitachi S-510, Hitachi-High Technologies Europe, Krefeld, Germany, equipped with a secondary electron detector) to gain detailed information on the distribution of curcumin within the pores of the paper matrix. For this, a horizontal section of the curcumin-loaded smartFilm tablets and their corresponding references (curcumin raw bulk material, physical mixture of paper and bulk curcumin, curcumin-loaded smartFilm granules, unloaded granules with 20% sucrose content) were sputter-coated with a thin layer of gold (10 mA for 1 min) using Edwards S150 Sputter Coater (Edwards Vacuum, Crawley, UK). An acceleration voltage of 5 kV was used to visualize the samples [24].

###### Determination of Crystalline State of Curcumin

X-ray diffraction (XRD) patterns were studied to evaluate the crystalline state of curcumin loaded within the smartFilm tablets. X-ray diffraction patterns from smartFilm tablets and from the according references (curcumin raw bulk material, physical mixture of paper and bulk curcumin, curcumin-loaded smartFilm granules, unloaded granules with 20% sucrose content) were recorded by using an X’Pert Pro MDP X-ray powder diffractometer (PANalytical/Philipps BV, Netherlands). The instrument was equipped with CuK α radiation (λ = 1.7903 Å) and operated at a voltage of 40 kV and 35 mA current. Samples were scanned at room temperature from 2θ = 10° to 2θ = 55° with a step of 0.03°/s.

###### Determination of Tablet Thickness and Mass Uniformity

The thickness of ten randomly selected tablets was determined using the IP67 ABS digimatic caliper (Mitutoyo, Kanagawa, Japan). Mass uniformity was evaluated according to the test method 2.9.5. of the European Pharmacopeia [22]. The test was performed on 20 randomly selected tablets. The tablets were weighed, then the average mass was calculated and compared to the mass of each individual tablet to determine the percentage deviation, followed by comparing the result to the European Pharmacopeia limits.

###### Determination of Friability

Twenty randomly selected tablets were used to evaluate the friability of the tablets according to test method 2.9.7. of the European Pharmacopeia using a friability tester equipped with an abrasion drum (PTF 10ER, Pharma Test Apparatebau AG, Hainburg, Germany) [22]. The tablets were weighed, then placed into a drum rotating at 25 rpm for 4 min. Following that, the tablets were removed, dedusted, reweighed and the percentage weight loss was calculated using the following equation:

(5) $$\%\;\mathrm{weight}\;\mathrm{loss}=100\left(\frac{W_{1}-W_{2}}{W_{1}}\right)\;$$

where *W*_1_ is the weight of the tablets before test and *W*_2_ is the weight of the tablets after test.

###### Resistance to Crushing

The crushing strength or hardness of a tablet is the force required to break down a tablet under compression and it was assessed according to the test method 2.9.8. of the European Pharmacopeia [22]. In this study, ten tablets were randomly selected, and each tablet was placed horizontally between the jaws of a hardness tester PTB 311E (Pharma Test Apparatebau AG, Hainburg, Germany), then the result was expressed in Newton (N) as mean value of the forces measured.

###### Disintegration

Disintegration of the tablets was evaluated in water according to the test method 2.9.1. of the European Pharmacopeia [22]. Six tablets were individually placed into the cavities of a disintegration tester PTZ-S (Pharma Test Apparatebau AG, Hainburg, Germany) that was operated at 37 °C ± 2 °C and the time required for complete tablet disintegration was recorded.

###### Content Uniformity

Curcumin content uniformity within the smartFilm tablets was investigated according to test method 2.9.6. [22]. Ten randomly selected curcumin-loaded smartFilm tablets were individually immersed in a mixture of 0.1 N hydrochloric acid (HCl) and 0.2% *w*/*w* sodium chloride (NaCl) solution containing 1% *w*/*w* SDS, under stirring, until complete disintegration of each tablet. 1% *w*/*w* SDS was added to ensure complete dissolution of the loaded curcumin. Samples were withdrawn and filtered, followed by discarding the initial ~6 mL of filtrate to ensure saturation of the filter. Following that, the amount of curcumin within each tablet was assessed spectrophotometrically at 425 nm using UV-vis spectroscopy (Multiskan™ GO, Thermo Fischer Scientific, Waltham, MA, USA) and a preconstructed calibration curve (5–10 mg/L).

###### Dissolution

The dissolution studies were carried out according to the test method 2.9.3. of the European Pharmacopeia in simulated gastric fluid without pepsin and in simulated intestinal fluid without pancreatin [22]. Six curcumin-loaded smartFilm tablets were individually placed into the vessels of a paddle apparatus PTWS 120D (Pharmatest, Apparatebau AG, Hainburg, Germany), which were filled with 750 mL of a mixture of 0.1 M HCl and 0.2% *w*/*w* NaCl solution containing 1% *w*/*w* SDS. The paddle speed was adjusted to 100 rpm and the temperature was kept at 37 °C ± 0.5 °C. Samples (10 mL) were taken after 0, 30, 45, 60, 75, 90, and 120 min and replaced with an equal volume (10 mL) of the fresh dissolution medium. After 2 h, 250 mL of 0.2 M tribasic sodium phosphate (TSP) buffer containing 1% *w*/*w* SDS, that has been equilibrated to 37 °C ± 0.5 °C, were added to the fluid in each vessel and within 5 min the pH was adjusted to 6.8. Samples (10 mL) were then taken after 1, 2, 4, 6, 10, and 18 h and replaced with an equal volume (10 mL) of a fresh mixture of 0.2 M TSP buffer containing 1% *w*/*w* SDS (pH 6.8), 0.1 M HCl and 0.2% *w*/*w* NaCl solution containing 1% SDS in a ratio of 1:3. All samples were filtered using a syringe filter with a pore size of 0.22 µm, then samples were analyzed regarding the amount of dissolved curcumin by using the UV-vis spectroscopy (cf. 2.2.4.7.) The same experiment was conducted by using physical mixture tablets that were produced using a mixture of unloaded paper granules with 20% sucrose content and the same content of curcumin powder.

##### 2.2.5. Determination of Intestinal Permeability

The physiology and the morphology of the porcine GIT bear a strong resemblance to that of the humans [25]. Therefore, the intestinal permeability of curcumin from the different formulations was determined on porcine intestines and the data analysis of permeated curcumin into the intestinal tissue was performed as previously described [26,27,28,29,30,31], with slight modifications. Fresh intact porcine gastrointestinal tracts were obtained from a local slaughterhouse and were used for the experiments within 2 h after slaughter. From the tissues obtained, small sections (approximately 15 cm in length) of intestinal tissue (i.e., 15–20 cm away from the pylorus) were isolated, dissected longitudinally and spread as a sheet. The intestinal sheets were gently wiped without affecting the villi structure and the pre-existing mucus and, subsequently 50 µL of the pre-digested formulations were applied onto it without any rubbing or mechanical stress. After 30 min permeation time, punch biopsies (Ø 15 mm) were obtained from the differently treated intestinal sections. The intestinal punches were immediately embedded in liquid embedding material (Tissue-Tek^®^ O.C.T.TM, Sakura Finetek Europe B.V., Alphen aan den Rijn, The Netherlands), and frozen at −20 °C until further use. Untreated intestinal samples were also biopsied and served as control. Each formulation was tested in triplicate and on different, independent intestinal tissues, i.e., on intestines from different pigs.

###### Pre-Digestion of the Formulations

Prior to the intestinal application, all formulations (physical mixture tablets, curcumin-loaded smartFilm tablets, commercial products I+II) were subjected to a pre-digestion (i.e., dissolution) procedure. In the first step, adequate amounts of each formulation, i.e., each containing an equivalent curcumin content (~30 mg), were incubated in 100 mL phosphate-buffered saline (PBS, pH 6.8, 37 °C) that contained 1% *w*/*w* SDS. The mixtures were stirred for 15 min (300 rpm, universal shaker SM-30 control (Edmund Bühler GmbH, Bodelshausen, Germany) and after this time, 50 µL aliquots were withdrawn and immediately applied onto the intestinal tissues (cf. 2.2.5.). In addition, similar samples, i.e., 50 µL from each pre-digested formulation, were withdrawn to analyze the exact amount of dissolved curcumin within these samples (Multiskan™ GO, Thermo Fischer Scientific, Waltham, MA, USA, 425 nm).

###### Digital Image Analysis

Curcumin possesses autofluorescence properties, that can be used to trace the permeation of it into tissues [26,27,28]. For this, the frozen intestinal biopsies were cut into vertical intestinal sections with a thickness of 40 µm by using a Frigocut-2700 cryomicrotome (Reichert-Jung, Wetzlar, Germany). The sections were placed onto microscopic slides and were subsequently examined by inverted epifluorescence microscopy (Olympus CKX53, Olympus Deutschland GmbH, Hamburg, Germany, equipped with Olympus DP22 colour camera, Olympus, Hamburg, Germany). The intensity of the fluorescent light source (130 W U-HGLGPS illumination system, Olympus Deutschland GmbH, Hamburg, Germany) was set to 50% and the exposure time was 50 ms. All samples were analyzed with a 40-fold magnification while using the DAPI HC filter block system (excitation filter: 340–390 nm, dichroic mirror: 410 nm, and emission filter: 420 nm (LP)). From each biopsy, at least 12 intestinal sections were obtained and from each Section 3 images were acquired. This resulted in a total of at least 108 images for each formulation tested (*n* = 3, 3 × 12 × 3 = 108).

In the next step, the images were subjected to digital image analysis using ImageJ software [32,33]. The first step was an automated threshold that subtracted the autofluorescence of the intestinal tissue (class II pixel) from the autofluorescence of the permeated curcumin (class I pixel, cf. Table S2). After application of the automated threshold, the remaining pixels within each image represent the amount of permeated curcumin as mean grey value/pixel (MGV/px), and thus surrogate the total amount of permeated (TAP) curcumin into the intestinal tissue semi-quantitatively [26,27,28,29,30,31]. The mean permeation depth of curcumin into the intestine was assessed from the thresholded images with the scale function of the software with the scale set to 0.55 pixel/µm.

##### 2.2.6. Statistical Analysis

Experiments were performed in triplicates and data were represented as mean ± standard deviation, unless otherwise noted. Descriptive statistics and statistical assessment of differences between the mean values were performed with JASP software version 16.2 (Universiteit van Amsterdam, Amsterdam, The Netherlands) [34]. After analyzing the data regarding normal distribution (Shapiro–Wilk test) and variance homogeneity (Levene’s test), data were subjected to one-way analysis of variance (ANOVA) or Kruskal-Wallis tests and adequate post hoc tests, such as Tukey’s, Games–Howell, Dunett and Dunn, were performed to compare the mean values with each other. In some cases, Student’s t-tests for pairwise comparison were performed and the correlation between the in vitro dissolution data with the data obtained from the ex vivo permeation data was determined by calculating the Spearman’s rank correlation coefficient ρ, respectively [34,35]. Differences between means were considered statistically significant if the *p*-value was <0.05.

## 3. Results and Discussion

### 3.1. Production and Characterization of Curcumin-Loaded smartFilm Granules

**Figure 2:**
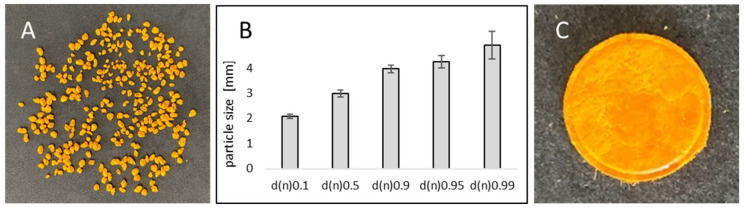
(**A**) Macroscopic image of smartFilm granules loaded with curcumin. (**B**) Numeric size distribution of the curcumin-loaded smartFilm granules. (**C**) Macroscopic image of a smartFilm tablet produced from the smartFilm granules loaded with curcumin.

Curcumin-loaded smartFilm granules with 20% sucrose content were successfully prepared (Figure 2A). The particle size (d(n) 0.5) was 3 mm ± 0.8 mm (Figure 2B), and the aspect ratio was 1.4. According to the literature, a value of 1.2 for the aspect ratio is usually appropriate to describe spherical particles [21]. This means the smartFilm granules produced in this study can be considered to possess a slightly elongated shape.

**Figure 3:**
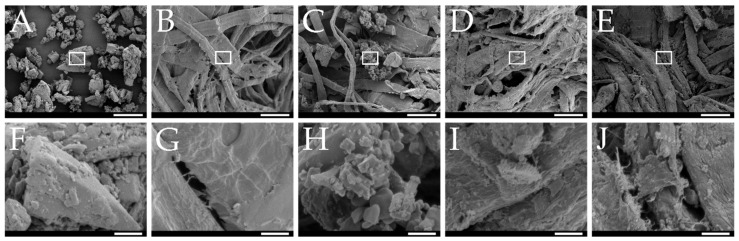
SEM micrographs of (**A**) pure curcumin, (**B**) unloaded paper granules, (**C**) physical mixture, (**D**) curcumin-loaded smartFilm granules, and (**E**) curcumin-loaded smartFilm tablets with the respective magnification (**F**–**J**) corresponding to the white square. Scale bars represent 60 µm in (**A**–**E**) and 7 µm in (**F**–**J**).

The bulk and tapped density of the smartFilm granules were 0.18 ± 0.0 and 0.21 ± 0.0, respectively. These values were significantly higher (Student’s *t*-test, *p* < 0.01) when compared to unloaded paper granules that were previously prepared using the same sucrose content (i.e., 20%) [11]. Hence, loading of curcumin increased the density of the granules. This is reasonable, because curcumin can be considered to be located in the pores of the cellulose matrix. This reduces the volume of the pores and thus increases the density of the formulation. The microscopic images obtained from scanning electron microscopy confirm this assumption. Curcumin bulk powder material is composed of small cubic particles (Figure 3A) and the non-loaded paper granules possess pores but contain no cubic particles (Figure 3B). The physical mixture shows that the cubic particles of the curcumin raw material are mixed with the paper matrix, i.e., both structures can be observed in this image (Figure 3C). For the smartFilm granules, no cubic particles are visible and the pores of the paper appear “filled” (Figure 3D). Thus, indicating that the curcumin was loaded into the pores of the paper in non-crystalline state.

The calculated Hausner’s ratio, Carr’s index and angle of repose were used as an indication of the flowability of the granules. Hausner’s ratio, Carr’s index and angle of repose values were 1.16 ± 0.0, 14.4 ± 0.0%, and 31° ± 0.0, respectively. According to the European Pharmacopeia, this indicates good flowability and compressibility of the prepared smartFilm granules [22]. These results were comparable to the results obtained previously from evaluating unloaded paper granules [11]. Thus, suggesting that the filling procedure of curcumin-loaded smartFilm granules, during high-speed tablet manufacturing, will operate efficiently with no major complications [36].

**Figure 4:**
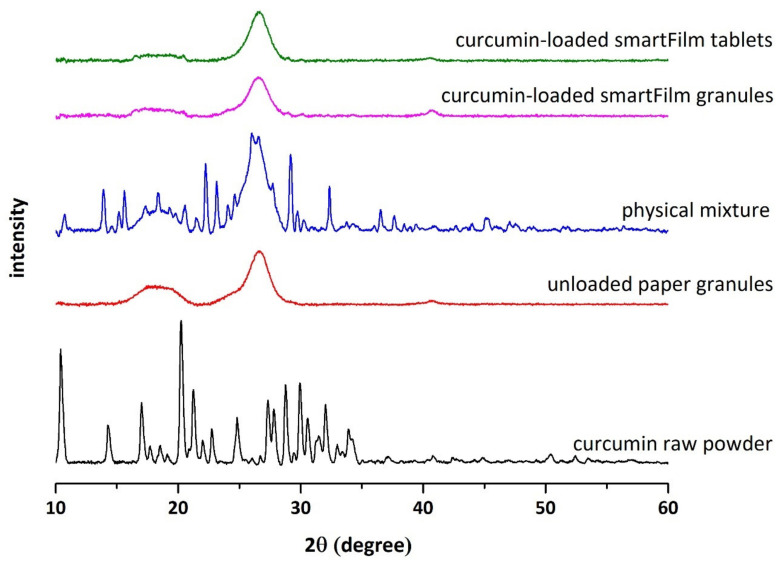
X-ray diffraction patterns of pure curcumin raw powder, unloaded paper granules, physical mixture, curcumin-loaded smartFilm granules, and curcumin-loaded smartFilm tablets.

X-ray data (Figure 4) revealed the typical reflexes for the crystalline curcumin bulk material [37]. The reflexes were also visible in the physical mixture that contained identical amounts of curcumin to that of the curcumin loaded smartFilm granules. In contrast, the X-ray pattern of the smartFilm granules showed no reflexes. Thus, suggesting that curcumin was embedded in the paper matrix of the smartFilms granules in amorphous state (Figure 4). This means, wet granulation of the smartFilms that contained curcumin in amorphous state [18] did not alter the crystalline state of the curcumin. Data of this part of the study therefore provided sufficient evidence that curcumin-loaded smartFilm granules can be transferred into smartFilm granules with sufficient pharmaceutical properties and showed that these smartFilm granules are able to maintain the amorphous state of the incorporated curcumin. The smartFilm granules obtained were therefore used as intermediate product for the production of curcumin-loaded smartFilm tablets (cf. 3.2.).

### 3.2. Production and Characterization of Curcumin-Loaded smartFilm Tablets

The compression of the curcumin-loaded smartFilm granules resulted in smooth, slightly porous and yellow-orange tablets with a height of 2.11 ± 0.05 mm (Figure 2C). Microscopic images from scanning electron microscopy showed the absence of crystalline curcumin particles within the tablets (Figure 3E), indicating that curcumin was embedded into the paper matrix in amorphous state. The data obtained from X-ray analysis confirmed this, i.e., no reflexes were found for the curcumin-loaded smartFilm tablets (Figure 4 upper).

In the next step, the produced tablets were evaluated regarding their pharmaceutical properties, i.e., mass uniformity, friability, hardness, disintegration time, content uniformity, and dissolution. The tablets fulfilled all criteria according to the European Pharmacopeia [22]. The average mass uniformity value was 2.0 ± 1.2% and the average weight of the produced tablets was 235 ± 0.005 mg. No tablet out of the 20 weighed tablets had an individual mass that differed by 7.5% from the average mass. The friability of the curcumin-loaded smartFilm tablets was found to be 0.086% and the European Pharmacopeia allows a maximum value of 1.0% [22]. Hence, the smartFilm tablets were within this limit. The crushing strength value was 115 ± 22 N, indicating a sufficient mechanical strength of the produced tablets. Interestingly, when compared to unloaded paper tablets [11], the data show an insignificant increase (Student’s *t*-test, *p* value > 0.05) of the force required to crush the curcumin-loaded tablets. It can be assumed that the effect is caused by the incorporation of curcumin into the pores of the paper matrix, which already resulted in an increased density of the smartFilm granules (cf. 3.1.).

Also regarding the disintegration time, results show that curcumin-loaded smartFilm tablets fulfilled the criteria according to the European Pharmacopeia, as all tablets disintegrated within 15 min [22]. It was also noticed that the disintegration time of curcumin-loaded tablets exhibited a slight increase when compared to the previously reported data that showed a faster disintegration time for unloaded-paper tablets with the same sucrose content [11]. This might be attributed to the hydrophobic nature of curcumin, which is abundantly loaded within the matrix of the paper. Hence, these data also show a trend towards a lower porosity of the smartFilm tablets, which results not only in an increased hardness of the tablets but also in a slower disintegration time.

The average amount of curcumin loaded within the produced tablets was 15.6 ± 0.5 mg. In addition, curcumin-loaded smartFilm tablets fulfilled the criteria according to the European Pharmacopeia with an average content uniformity value of 97.6 ± 2.0%, and no tablet out of the ten examined tablets exhibited a content value that differed by 15% from the average content [22].

**Figure 5:**
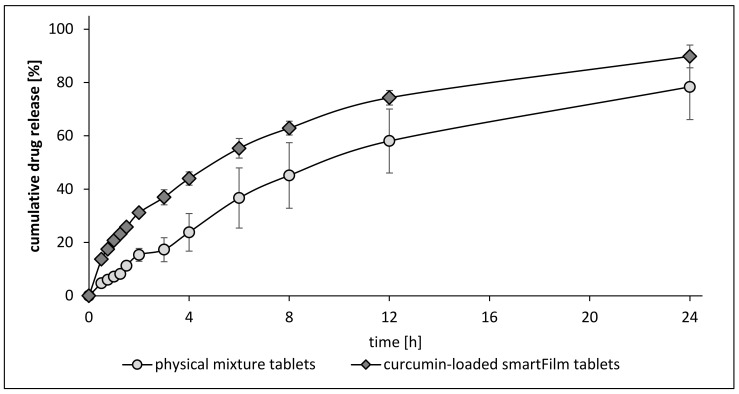
The dissolution profiles of curcumin from physical mixture tablets and curcumin-loaded smartFilm tablets (explanations and details cf. text and Figure S1).

For the cumulative release profiles of curcumin, significant differences between the physical mixture tablets and curcumin-loaded smartFilm tablets were found for all time points (Figure 5).

The differences in the dissolution velocity were most pronounced in the beginning. For example, after 1 h, the amount of released curcumin from the smartFilm tablets was about 20.8% ± 1.1% and was only 7.2% ± 1.8% from the physical mixture tablets (Figure 5). Within 24 h, curcumin-loaded tablets released 89.8% ± 4.3%, compared to 78.3% ± 12.3% of curcumin that was released from the physical mixture tablets (Figure 5).

The results demonstrate that the differences in dissolution rate between smartFilm tablets and physical mixture tablets changed over time, i.e., decreased over time. The increase was about three-fold in the beginning (30 min–1 h) and was about two-fold between 2 h and 4 h dissolution time. After 12 h, the amount of dissolved curcumin was about 30% higher for the smartFilm tablets and about 15% higher after 24 h dissolution time (Figure 5). The findings therefore prove that the dissolution rate of curcumin can be improved with smartFilm tablets and show that the dissolution rate increasing effect of the smartFilms is most pronounced at early time points. The fast dissolution at early time points can be considered to be advantageous, because not only an improved but also a fast dissolution of poorly soluble drugs is considered to be important to improve their oral bioavailability [38,39,40].

### 3.3. Determination of Intestinal Permeability

**Figure 6:**
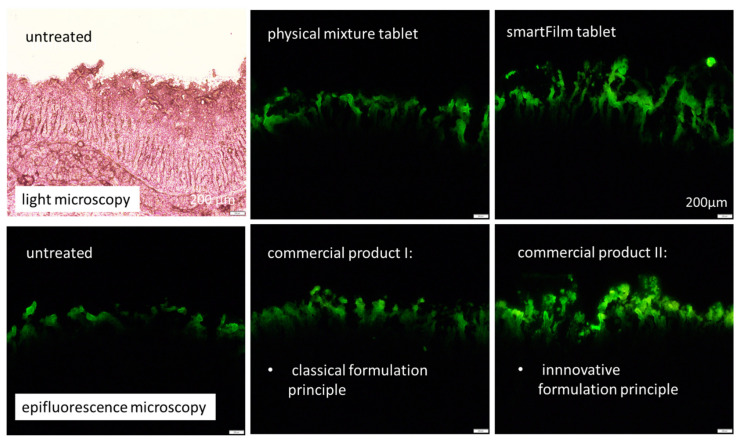
Epifluorescence microscopic images (40-fold magnification) of porcine intestinal sections treated with different curcumin-loaded formulations, as compared to the untreated intestinal tissue. In addition, an image of the intestinal tissue with similar magnification taken with light microscopy is shown to visualize the morphology of the intestinal tissue used for the study (**upper left**). Scale bars correspond to 200 µm.

The small intestine is the main absorption site for the majority of orally administered drugs [41]. The intestinal epithelium is arranged into crypts and villi and is covered by a mucus layer, that acts as a barrier for the diffusion of drugs to the underlying epithelium [42,43]. Due to the complexity of the intestinal tissue, simple in vitro release data cannot cover all aspects that occur during the absorption of drugs into the gut. Therefore, besides in vivo studies that cannot always be performed, ex vivo intestinal models that are able to closely mimic the physiological conditions of the small intestine, are important tools to predict the intestinal permeability of drugs from different formulations [44]. In this study, the porcine intestines were treated with the different formulations and the permeation of curcumin into the intestinal tissue was inspected with epifluorescence microscopy (Figure 6).

**Figure 7:**
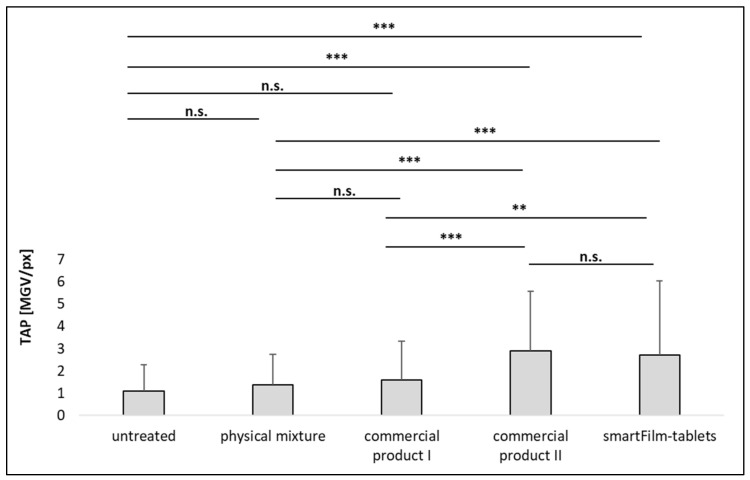
Permeated amount of curcumin from different formulations. The total amount permeated (TAP, MGV/px) is a semi-quantitative parameter that surrogates the intestinal permeability, i.e., oral bioavailability, of curcumin (n.s.—nonsignificant, ** *p* < 0.01, *** *p* < 0.001).

**Figure 8:**
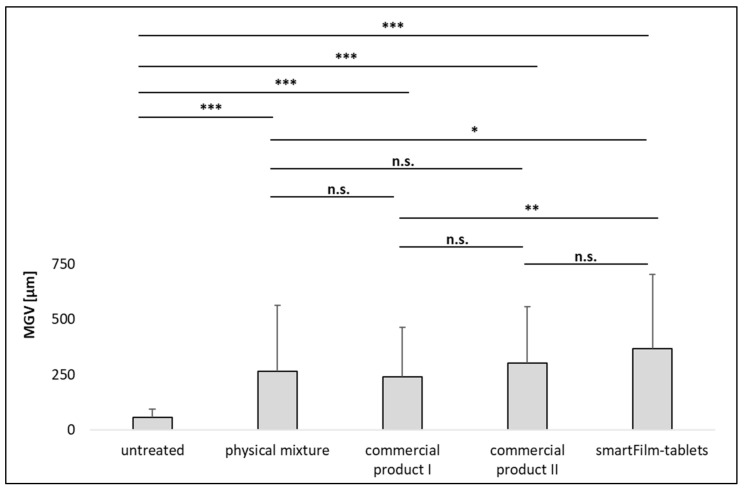
Mean permeation depth of curcumin from different formulations. (n.s.—nonsignificant, * *p* < 0.05, ** *p* < 0.01, *** *p* < 0.001).

The images show different permeation profiles of curcumin from the different formulations and show a trend towards an improved permeation of curcumin from the smartFilm tablets and the commercial product II (Figure 6). The trends observed from the images became clearer and proved being significant after digital image analysis of the images that assessed the total amount of permeated curcumin (TAP) and the mean permeation depth (MPD), (Figure 7 and Figure 8).

No significant differences in the signal intensity (MGV/px) were found between untreated intestine, the physical mixture tablet, and the commercial product I (Figure 7). However, the permeation depth was significantly higher for the physical mixture tablet and the commercial product I when compared to the untreated tissue (Figure 8). Data indicate that both formulations can be considered to possess a very poor intestinal permeability for curcumin. This was expected, because both formulations contain the curcumin as powder, which is known to possess poor solubility and poor intestinal permeability [15].

Commercial product II, the micellar curcumin, which is considered to result in an optimal oral bioavailability and intestinal absorption of curcumin (cf. 1., [16]), resulted in significantly higher amounts of permeated curcumin than the physical mixture tablet and commercial product I (Figure 7). However, the permeation depth was not altered when compared to the physical mixture tablet and commercial product I (Figure 8).

The smartFilm tablets resulted in similarly high amounts of permeated curcumin as commercial product II (Figure 7). Interestingly, in comparison to the physical mixture and commercial product I, it was found that the permeation depth was significantly enhanced (+38% and +52%, respectively) when curcumin was applied with the smartFilms. The increase in permeation depth was 22% but nonsignificant in comparison to commercial product II (Figure 8).

With this, the smartFilm tablets can be considered to yield comparable intestinal permeation values for curcumin to that of the micellar curcumin, with a slight trend towards a deeper permeation of curcumin. The deeper permeation from the smartFilm tablets is reasonable, because the pre-digestion procedure fully disintegrated the smartFilm tablet, but left behind some pieces of paper. The paper adhered to the gut and can be considered to have caused a locally higher concentration gradient for curcumin between the smartFilm and the intestine, which then promoted a deeper permeation into the intestinal tissue. Improved permeation via a locally high concentration gradient was previously shown for particles and smartFilms that were applied on skin [18,45] and it is very likely that similar effects also occur in the gut. However, more research is needed to investigate and understand this observation in detail.

**Figure 9:**
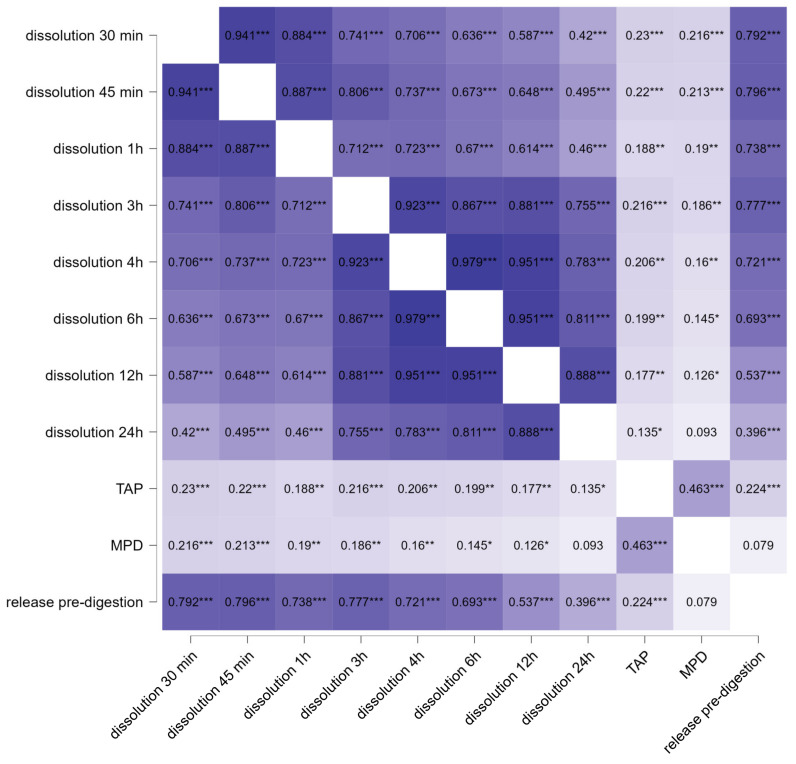
Heatmap of Spearman’s rank correlation coefficients ρ that assess the relationship between the in vitro dissolution data and the ex vivo permeation data for the physical mixture and the smartFilm tablets. * *p* < 0.05, ** *p* < 0.01, *** *p* < 0.001.

In the last part of the study the relationship between the in vitro dissolution data (cf. Figure 5) and the ex vivo permeation data (TAP and MPD) was assessed for the physical mixture and the smartFilm tablets (Figure 9). In addition, to gain more detailed information on the in vitro ex vivo correlation, the amount of released curcumin, that was assessed from all different formulations after the in vitro pre-digestion step and immediately prior to the application onto the intestinal tissues, was correlated to the ex vivo permeation data for all formulations tested (cf. Figure 9, right).

The correlation of the data from physical mixture and smartFilm tablets show significant relations between the in vitro data and the ex vivo data (Figure 9). A very strong correlation (>0.8) was found between the dissolution data after 30 and 45 min of dissolution. With longer dissolution times the correlation coefficient decreased to <0.5 after 24 h, which is still considered to be a relatively strong correlation [46]. However, the decrease in correlation coefficient indicates that the release of curcumin is not following a constant function.

Reasons for this might be, for example, a partial oversaturation of the system and subsequent re-crystallization of the curcumin. Another possibility is the non-linear release of the curcumin from the paper matrix of the smartFilms (cf. Figure 5), which were recently shown to follow a super case II release kinetics, which is caused by the changes that occur after the wetting of the paper matrix [18].

In this study, the formulations were applied onto the intestinal tissue after a 15 min pre-digestion step. This is closest to the 30 min time point for the in vitro dissolution data. The correlation coefficient between the in vitro data 30 min is 0.233 (*p* < 0.001) for the TAP and 0.216 (*p* < 0.001) for the MPD (Figure 9), which represents a moderate correlation according to Rea and Parker [46]. The correlation coefficient decreases, and the *p*-values increase with increasing dissolution time, indicating that the in vitro–ex vivo correlation between dissolution data and ex vivo model declines for longer dissolution times. This trend was expected and points towards a high sensitivity of the intestinal model because the dissolution of curcumin is nonlinear. Therefore, a good in vitro ex vivo correlation can only be yielded if similar dissolution/pre-digestion times are used for the correlation of the data.

For the comparison between the in vitro and ex vivo data for all formulations tested, the correlation between curcumin released after the pre-digestion and the amount of permeated curcumin into the intestine was calculated. The resulting Spearman’s rank correlation coefficient ρ was 0.224 (*p* < 0.001), which also indicates a moderate in vitro ex vivo correlation for the total amount of permeated drug [46]. However, the correlation coefficient was only 0.079 (*p* = 0.05) for the MPD, indicating no in vitro ex vivo correlation between the in vitro dissolution data and the ex vivo permeation depth. This might be explained by the deeper permeation depth of the curcumin from the smartFilm tablets, which was probably caused by the parts of paper that adhered to the intestinal tissue (cf. above). To prove this theory, the Spearman’s rank correlation coefficient was re-calculated while excluding the data obtained from the smartFilm tablet formulation. The resulting correlation coefficient was 0.128 (*p* = 0.01), which indicates a weak but significant in vitro ex vivo correlation [46]. The results further substantiate the theory that the smartFilm tablets can cause an improved permeation depth of curcumin via a locally high concentration gradient that resulted from the adherence of pieces of paper to the intestinal tissue.

According to Kinam Park and co-workers BCS class IV drugs are very unlikely to yield strong in vitro ex vivo correlations between dissolution profiles and in vivo permeation, whereas good and very strong in vitro ex vivo correlations can be established for BCS class II drugs [40]. In most cases, curcumin is considered a BCS class IV drug [47,48]. However, sometimes it is also referred to be a BCS-class II drug [49]. Thus, while considering curcumin as an intermediate drug substance with BCS class II and IV properties, the in vitro ex vivo correlations found in this study become very reasonable. Nevertheless, when compared to the in vitro data, the ex vivo data obtained here allowed for a more detailed investigation of the effect of the different formulation principles on the intestinal permeation of curcumin. Therefore, the use of an intestinal ex vivo model can be recommended as a simple and cost-efficient approach to judge the intestinal permeation efficacy of drugs from different formulations in early formulation development. The ex vivo data obtained in this study could clearly discriminate between the formulations possessing poor or good intestinal permeation properties for curcumin. Thus, providing a base for an efficient development of optimized formulations with excellent oral bioavailability for curcumin.

## 4. Conclusions

The study showed that drug-loaded smartFilms can be transferred into smartFilm granules and smartFilm tablets. Results also showed that the curcumin-loaded smartFilm granules and smartFilm tablets maintained the amorphous state of the incorporated drug. Hence, wet granulation was found to be a feasible approach for the production of smartFilm granules from which smartFilm tablets can be compressed in industrial, large scale. The resulting tablets fulfill the criteria according to the European Pharmacopeia regarding resistance to crushing, friability, content uniformity, mass uniformity, and disintegration time. The formulation of curcumin in smartFilm tablets resulted in an improved dissolution rate and enhanced intestinal permeability when compared to a physical mixture tablet. The intestinal permeability of curcumin from a marketed product that contained curcumin as raw powder was not significantly higher than the physical mixture but was at least two-fold higher for a commercial product that contained micellar curcumin. A two-fold higher intestinal permeability was also found for the smartFilm tablets. Between the commercialized micellar curcumin (for which previous in vivo studies already demonstrated a superior oral bioavailability in comparison to other innovative formulation principles, e.g., liposomes, phytosomes, submicron crystals, or cylcodextrines) and the smartFilm tablets no significant differences in the total amount of permeated curcumin were found. However, a trend towards a deeper permeation of the curcumin from the smartFilms was found. The effect was significant, thus rendering the smartFilm tablets to be the most effective formulation for the intestinal permeation of curcumin. Based on the results it can therefore be concluded that smartFilm tablets are an industrially feasible formulation approach for improved oral delivery of poorly soluble drugs.

## Data Availability

Not applicable.

## Supplementary Materials

The following supporting information can be downloaded at: https://www.mdpi.com/article/10.3390/pharmaceutics14091918/s1, Table S1: Macro used for the determination of the Feret’s diameter, with a scale set to 20 px/mm.; Table S2: Macro used for the automated threshold to subtract the autofluorescence of the intestinal tissue from the fluorescence of the permeated curcumin. Figure S1: The dissolution profiles of curcumin from physical mixture tablets and curcumin-loaded smartFilm tablets. * *p* < 0.05, ** *p* < 0.01, *** *p* < 0.001.
