# Supplementary material for: SmartFilm Tablets for Improved Oral Delivery of Poorly Soluble Drugs
Source: Pharmaceutics. 2022 Sep 10;14(9):1918. doi: 10.3390/pharmaceutics14091918 (PMC9500710; doi:10.3390/pharmaceutics14091918)
Supplement: Supplementary file 1 [file pharmaceutics-14-01918-s001.zip › pharmaceutics-1880347-supplementary.pdf]

pharmaceutics

Article

smartFilm tablets for improved oral delivery of poorly soluble drugs

Ayat Abdelkader [1,2], Eduard Preis [1] and Cornelia M. Keck [1,*]

[1] Department of Pharmaceutics and Biopharmaceutics, Philipps-Universität Marburg, Robert-Koch-Str. 4, 35037 Marburg, Germany
[2] Assiut International Center of Nanomedicine, Al-Rajhi Liver Hospital, Assiut University, 71515 Assiut, Egypt
* Correspondence: cornelia.keck@pharmazie.uni-marburg.de; Tel.: 49 6421 282 5881

Supplementary Materials:

Table S1. Macro used for the determination of the Feret`s diameter, with a scale set to 20 px/mm.

```
run("8-bit");
setAutoThreshold("Default");
//run("Threshold...");
//setThreshold(0, 180);
setOption("BlackBackground", false);
run("Convert to Mask");
run("Analyze Particles...", "size=0.09-Infinity show=Masks display");
```

Table S2. Macro used for the automated threshold to subtract the autofluorescence of the intestinal tissue from the fluorescence of the permeated curcumin.

```
// Color Thresholder 1.53k
// Autogenerated macro, single images only!
min=newArray(3);
max=newArray(3);
filter=newArray(3);
a=getTitle();
run("RGB Stack");
run("Convert Stack to Images");
selectWindow("Red");
rename("0");
selectWindow("Green");
rename("1");
selectWindow("Blue");
rename("2");
min[0]=0;
max[0]=0;
filter[0]="stop";
```

```
min[1]=75;
max[1]=255;
filter[1]="pass";
min[2]=0;
max[2]=0;
filter[2]="stop";
for (i=0;i<3;i++){
   selectWindow(""+i);
   setThreshold(min[i], max[i]);
   run("Convert to Mask");
   if (filter[i]=="stop")    run("Invert");
}
imageCalculator("AND create", "0","1");
imageCalculator("AND create", "Result of 0","2");
for (i=0;i<3;i++){
   selectWindow(""+i);
   close();
}
selectWindow("Result of 0");
close();
selectWindow("Result of Result of 0");
rename(a);
// Colour Thresholding-------------
run("Invert");
```

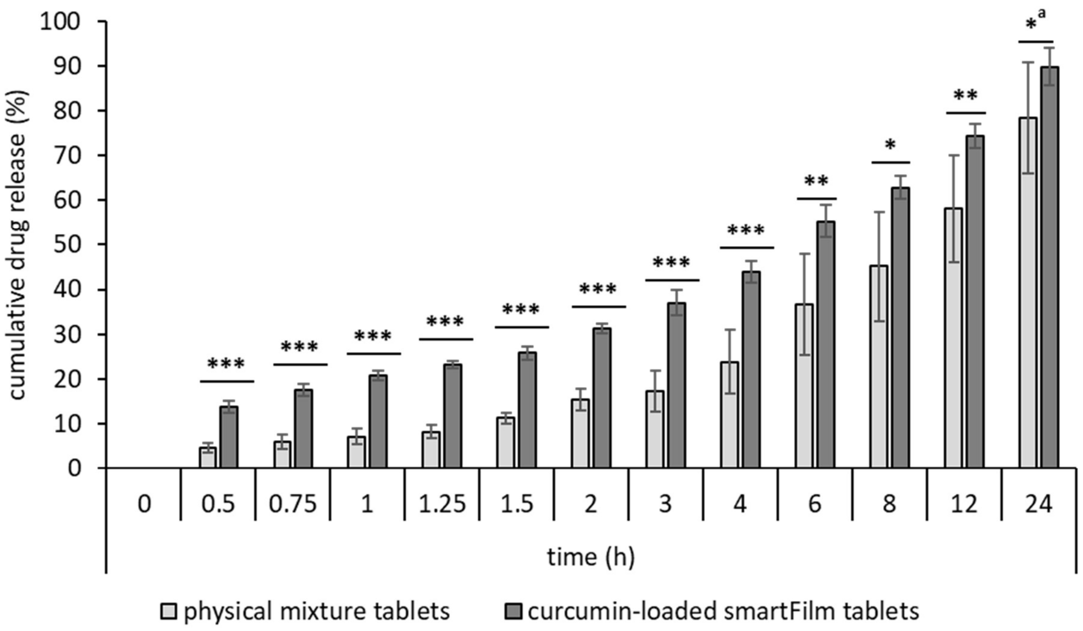

[a] one-tailed t-test

Figure S1. The dissolution profiles of curcumin from physical mixture tablets and curcumin-loaded smartFilm tablets. * $p < 0.05$, ** $p < 0.01$, *** $p < 0.001$.
